# Smooth muscle protein 22α-Cre recombination in resting cardiac fibroblasts and hematopoietic precursors

**DOI:** 10.1038/s41598-022-15957-2

**Published:** 2022-07-07

**Authors:** Shinya Ikeda, Sachiko Sugioka, Takeshi Kimura, Noboru Ashida

**Affiliations:** 1 Department of Cardiovascular Medicine, Graduate School of Medicine, Kyoto University, 54 Kawahara-cho, Shogoin, Sakyo-ku, Kyoto, 606-8507 Japan; 2 Hirakata Kohsai Hospital, Osaka, Japan

**Keywords:** Cardiovascular models, Genetic engineering, Biotechnology

## Abstract

The Cre-loxP system has been widely used for cell- or organ-specific gene manipulation, but it is important to precisely understand what kind of cells the recombination takes place in. Smooth muscle 22α (SM22α)-Cre mice have been utilized to alter genes in vascular smooth muscle cells (VSMCs), activated fibroblasts or cardiomyocytes (CMs). Moreover, previous reports indicated that SM22α-Cre is expressed in adipocytes, platelets or myeloid cells. However, there have been no report of whether SM22α-Cre recombination takes place in nonCMs in hearts. Thus, we used the double-fluorescent Cre reporter mouse in which GFP is expressed when recombination occurs. Immunofluorescence analysis demonstrated that recombination occurred in resting cardiac fibroblasts (CFs) or macrophages, as well as VSMCs and CMs. Flow cytometry showed that some CFs, resident macrophages, neutrophils, T cells, and B cells were positive for GFP. These results prompted us to analyze bone marrow cells, and we observed GFP-positive hematopoietic precursor cells (HPCs). Taken together, these results indicated that SM22α-Cre-mediated recombination occurs in resting CFs and hematopoietic cell lineages, including HPCs, which is a cautionary point when using SM22α-Cre mice.

## Introduction

The Cre-loxP system enables us to edit genes of interest in a tissue- or cell-specific manner. However, it goes without saying that researchers should understand precisely which kind of cells are subjected to recombination. In cardiovascular research, Smooth muscle 22α (SM22α)-Cre has been widely used to manipulate genes in vascular smooth muscle cells (VSMCs), activated fibroblasts and cardiomyocytes (CMs)^1–4^. Moreover, SM22α-Cre has been reported to be expressed in adipocytes, platelets and multiple lineages of myeloid cells^5,6^. However, there has been no report of whether SM22α-Cre recombination takes place in nonCMs in hearts. We designed a study to answer this question.

In this study, we used double-fluorescent Cre reporter mice^7^ mated with SM22α-Cre (SMmTmG) mice and analyzed relevant indices by immunofluorescence and flow cytometry.

## Result

### SM22α-Cre is expressed in quiescent cardiac fibroblasts (CFs) and macrophages in adult and embryonic hearts

**Figure 1:**
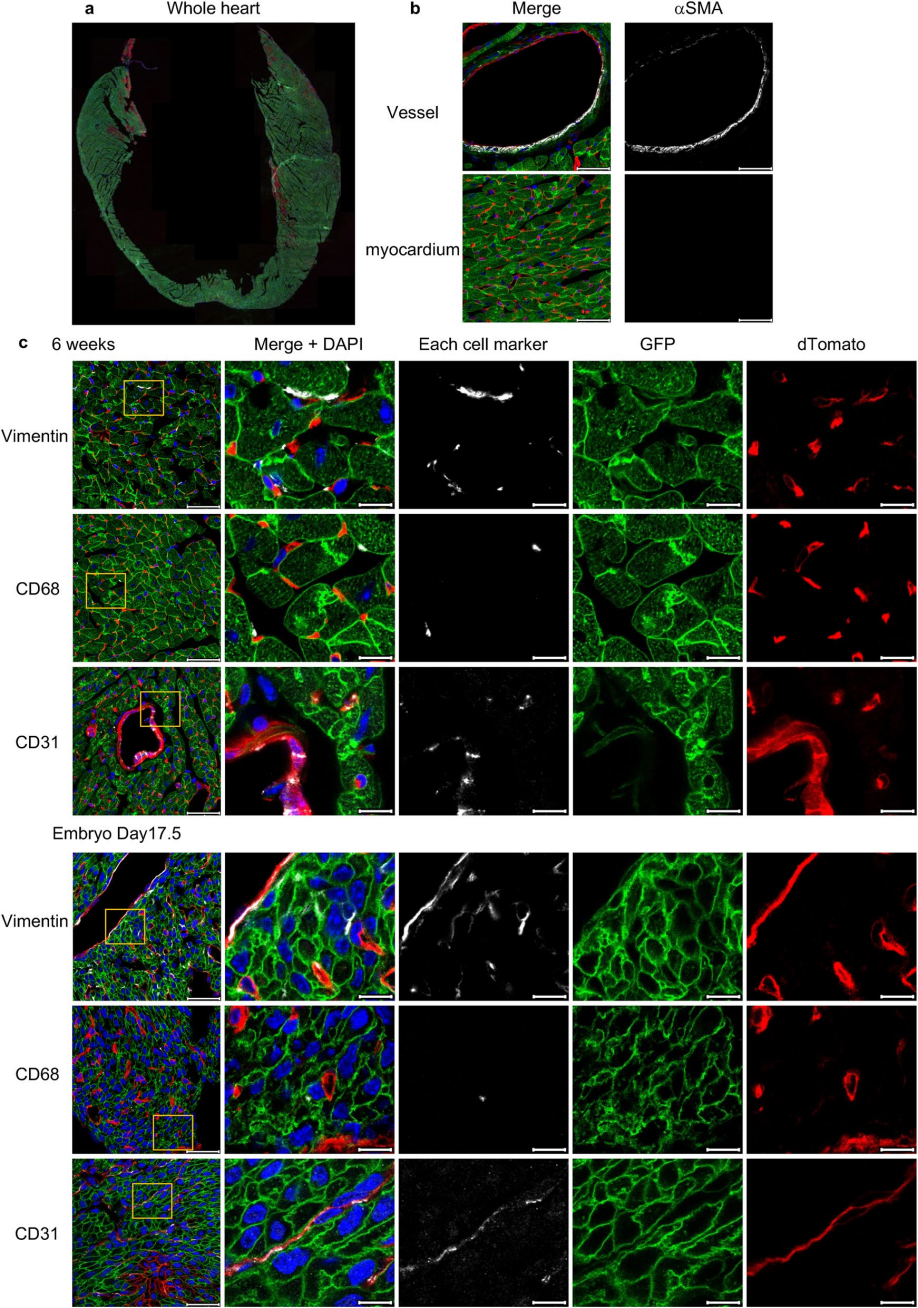
Immunofluorescence analysis of the hearts of SMmTmG mice. (**a**) A representative image of the whole heart without any staining. Approximately 90% of CMs expressed GFP. Original magnification:  ×5. (**b**) The expression of αSMA in cardiac vessels and the myocardium. VSMCs expressed GFP. Original magnification:  ×63. Scale bars: 40 μm. (**c**) The expression of the cell-specific markers, GFP and dTomato (left panels) and details (right 4 panels) in adult and embryonic hearts. There were some GFP+ CFs (Vimentin+) and GFP+ macrophages (CD68+), but no GFP+ endothelial cells (CD31+). Original magnification: × 63. Scale bars: 40 μm (original images) or 10 μm (details).

We evaluated in which types of cells SM22α-Cre recombination takes place in adult and embryonic murine steady-state hearts. Similar to a previous study^1^, VSMCs and approximately 90% of CMs expressed GFP (Fig. 1a,b). Surprisingly, there were many GFP and Vimentin positive cells even though there were no myofibroblasts in murine steady-state hearts^8^ (Fig. 1b,c). This finding suggested that SM22α-Cre was expressed in resting CFs. Moreover, GFP was detected in some macrophages (CD68+) (Fig. 1c). However, there were no GFP-positive endothelial cells (CD31+) (Fig. 1c), which are the most numerous cell type in the murine heart.

### Flow cytometric analysis of SM22α-Cre expression in hearts

**Figure 2:**
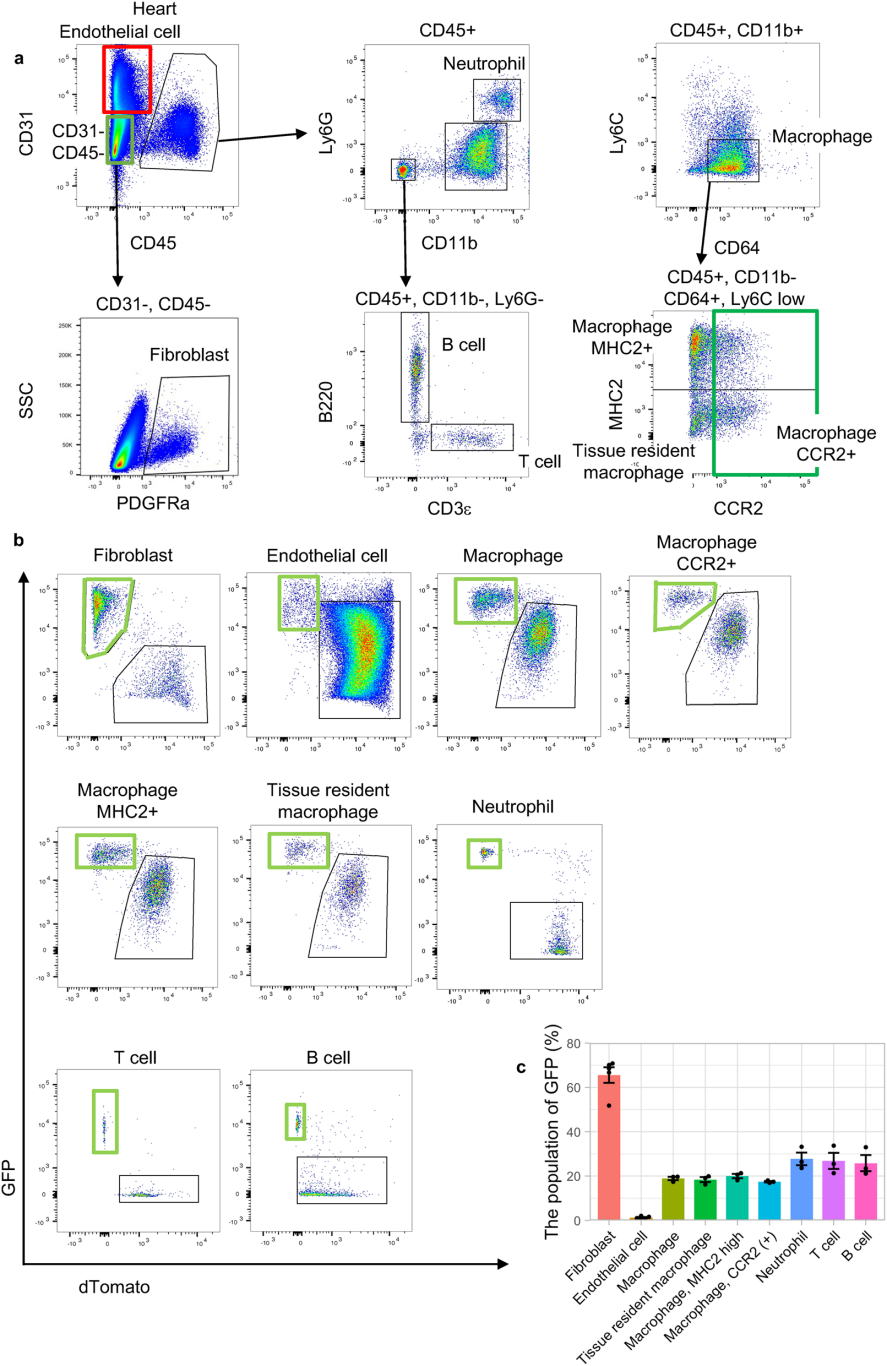
Flow cytometric analysis of nonCMs in SMmTmG mice. (**a**) The representative gating schema of murine hearts. (**b**) GFP expression in each cell type in the heart. (**c**) Summary of GFP expression in the heart (fibroblasts and endothelial cells: n = 5, others: n = 3).

We were interested in the precise GFP percentages among specific cell types, such as fibroblasts and macrophages. Flow cytometric analysis of adult murine hearts revealed that fibroblasts (CD31−, CD45−, PDGFRa+) (65.6 ± 3.5%) had a GFP-positive group, while very few endothelial cells (CD31+ and CD45−) (1.3 ± 0.2%) expressed GFP, which was in agreement with the immunofluorescent analysis (Fig. 2a–c). Detailed investigation of the immune cells revealed that some GFP-positive populations were present among macrophages (CD45+, CD11b+, CD64+, Ly6c low) (18.9 ± 0.8%) and neutrophils (CD45+, CD11b+, Ly6G+) (27.7 ± 2.9%) as described in a previous paper^5^. Furthermore, some cardiac resident macrophages (CD45+, CD11b+, CD64+, Ly6c low, CCR2−, MHC2 low) (18.4 ± 1.1%) and MHC2 high macrophage (CD45+, CD11b+, CD64+, Ly6c low, CCR2-, MHC2 high) (20.0 ± 1.0%), both of which are derived from the yolk sac or fetal liver^9,10^, expressed GFP (Fig. 2a–c). Surprisingly, among T cells (CD45+, CD11b−, CD3ε+) (26.8 ± 3.6%) and B cells (CD45+, CD11b−, B220+) (25.8 ± 3.6%), there were GFP-positive populations, unlike in a previous paper^5^ (Fig. 2a–c).

### Flow cytometric analysis of SM22α-Cre expression in peripheral blood and bone marrow

**Figure 3:**
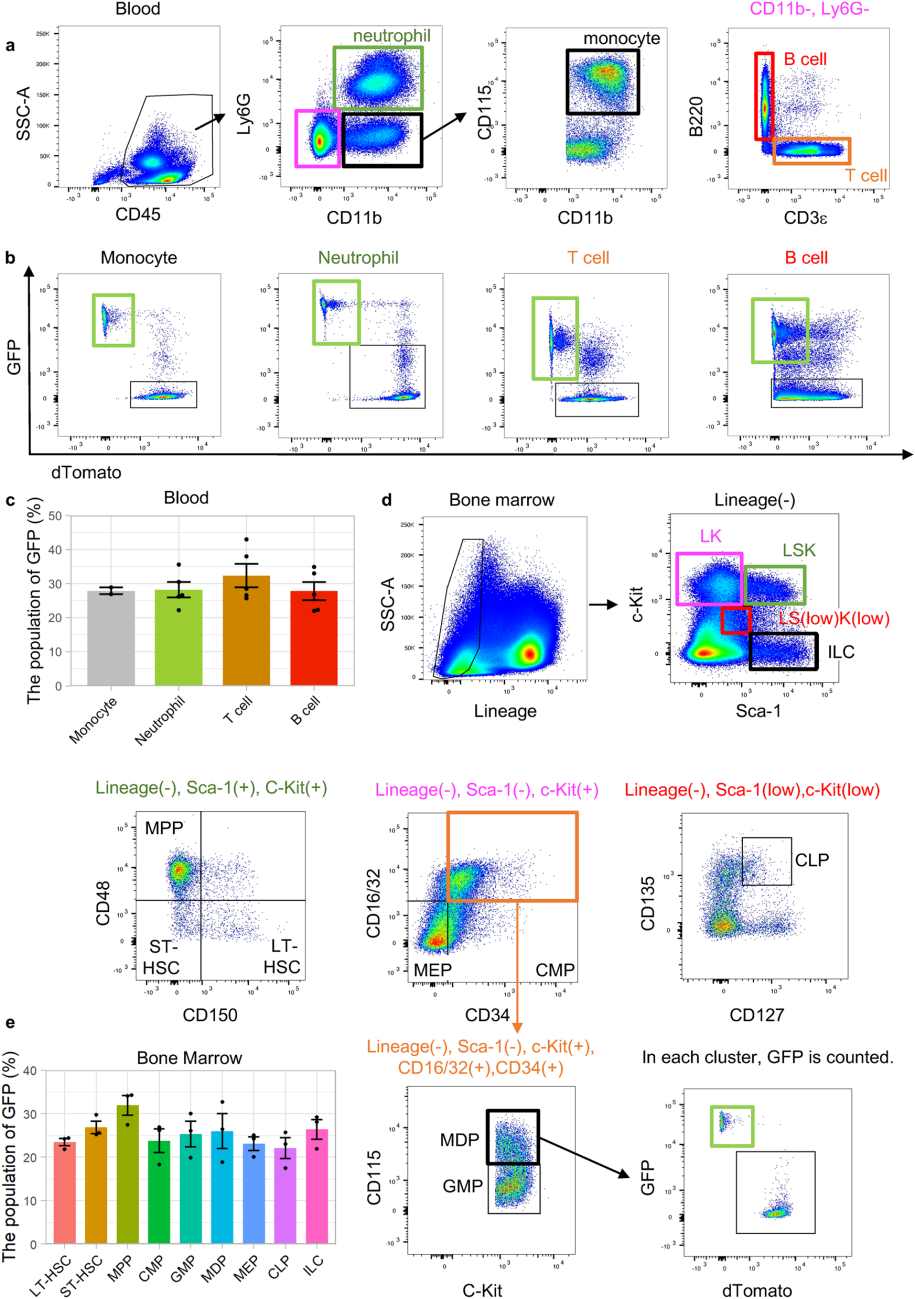
Flow cytometric analysis of the peripheral blood and bone marrow of SMmTmG mice. (**a**) The representative gating schema in the peripheral blood. (**b**) GFP expression in each cell type in the peripheral blood. (**c**) Summary of GFP expression in peripheral blood (monocytes: n = 2, others: n = 5). (**d**) The representative gating schema in the bone marrow. *LT-HSC* phenotypically defined putative long-term hematopoietic stem cell, *ST-HSC* phenotypically defined putative short-term hematopoietic stem cell, *MPP* multipotent progenitor, *CMP* common myeloid progenitor, *GMP* granulocyte macrophage progenitor, monocyte-macrophage dendritic cell progenitor, *MEP* megakaryocyte-erythrocyte progenitors, *CLP* common lymphoid progenitor, *ILC* innate lymphoid cell. (**e**) Summary of GFP expression in the bone marrow (n = 3).

We assessed whether SM22α-Cre was expressed in adult murine peripheral blood and bone marrow because we identified GFP-positive groups among T cells and B cells in the heart. Consistent with the results obtained in the heart, GFP was detected in monocytes (27.9 ± 1.0%), neutrophils (28.2 ± 2.3%), T cells (32 ± 3.4%) and B cells (27.8 ± 2.7%) in peripheral blood (Fig. 3a–c). Surprisingly, phenotypically defined putative long-term hematopoietic stem cells (LT-HSCs, Lineage−, c-Kit+, Sca1+, CD150+, CD48−) (23.5 ± 0.8%) and short-term HSCs (ST-HSCs, Lineage-, c-Kit+, Sca1+, CD150−, CD48−) (26.8 ± 1.4%), multipotent progenitors (MPPs, Lineage−, c-Kit+, Sca1+, CD150-, CD48 +) (31.9 ± 2.3%)^11^, downstream myeloid lineages, such as common myeloid progenitors (CMPs, Lineage−, c-Kit+, Sca1−, CD34+, CD16/32−) (23.8 ± 2.7%), granulocyte macrophage progenitors (GMPs, Lineage-, Sca-1−, c-Kit+, CD34+, CD16/32+, CD115−) (25.3 ± 2.9%), monocyte-macrophage dendritic cell progenitors (MDPs, Lineage−, Sca-1−, c-Kit+, CD34+, CD16/32+, CD115+) (26.0 ± 4.0%), and megakaryocyte-erythrocyte progenitors (MEPs, Lineage-, Sca-1−, c-Kit+, CD34−, CD16/32−) (23.1 ± 1.6%)^12^, and the lymphoid lineages, including common lymphoid progenitors (CLPs, Lineage-, Sca-1 low, c-Kit low, CD135+, CD127+) (22.1 ± 2.4%), innate lymphoid cells (ILCs, Lineage−, Sca-1+, c-Kit−) (26.4 ± 2.3%)^13^ had GFP-positive populations (Fig. 3d,e and Supplementary Fig. 1).

### In vitro, SM22α-Cre was expressed in CFs, but not in bone marrow-derived macrophages (BMDMs)

We investigated whether cells isolated from SM22α-Cre mice were useful for editing genes in vitro because the efficiency of gene editing, such as that mediated by a lentivirus, still has room for consideration.

**Figure 4:**
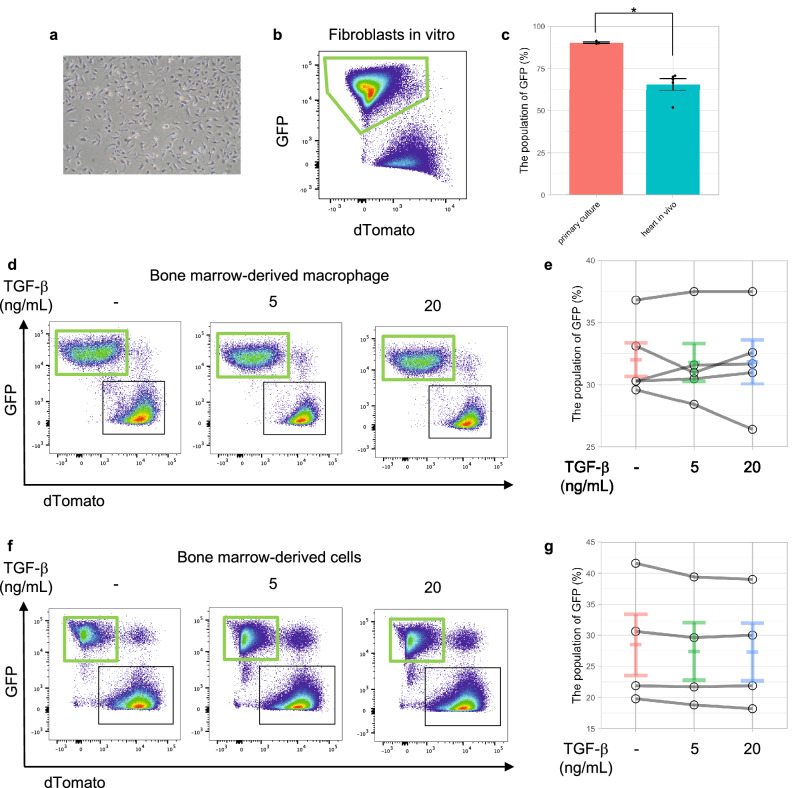
SM22α-Cre recombination in vitro. (**a**) A representative bright field image of cultured fibroblasts. (**b**) GFP expression in fibroblasts in vitro. (**c**) GFP expression in fibroblasts in vitro (n = 3) and in vivo (n = 5). *p value < 0.01, evaluated by Student’s t test. (**d**) GFP expression in BMDMs with TGF-β stimulation. (**e**) No additional SM22α-Cre recombination in BMDMs with TGF-β stimulation, which increases SM22α expression. (**f**) GFP expression in bone marrow-derived cells with TGF-β stimulation. (**g**) No additional SM22α-Cre recombination in bone marrow-derived cells during differentiation from phenotypically defined putative HSCs to macrophages with TGF-β stimulation.

Almost all isolated CFs (90.3 ± 0.5%) can become GFP + cells by being seeded on a culture dish because fibroblasts become myofibroblasts on a culture dish^14^ (Fig. 4a–c). However, TGF-β stimulation can upregulate Tagln (the SM22α gene) and Acta2 (the αSMA gene) expression in BMDMs, but the expression levels were so much lower than those in fibroblasts that SM22α-Cre recombination could not be used in BMDMs (Fig. 4d,e and Supplementary Fig. 2).

Furthermore, we analyzed whether differentiation facilitates SM22α-Cre recombination. Flow cytometric analysis showed that differentiation had no effect on recombination under TGF-β stimulation (Fig. 4f,g).

## Discussion

SM22α-Cre recombination has not been fully assessed in murine hearts, although SM22α-Cre has been widely used to alter VSMCs, activated fibroblasts and CMs in cardiovascular research^2–4^. This study offers further insights into the target of SM22α-Cre and how to lead recombination in some cells. First, SM22α-Cre recombination occurs in 65% of resting CFs. Second, SM22α-Cre recombination occurs in cardiac T and B cells, in addition to myeloid cells. Third, recombination also takes place in putative HSCs. Finally, CFs have the capacity to express SM22α-Cre. However, TGF-β, which is well known to induce Tagln^15–17^, cannot induce SM22α-Cre recombination in BMDMs.

One important finding in the present study is that there are some GFP-positive populations among resting CFs from embryos. SM22α is a well-known marker of activated fibroblasts^18^. In vivo, SM22α-Cre cannot be used to target only activated CFs, but could be used to manage quiescent CFs. As stated in previous papers^5,6,19^, we recommend other Cre recombination systems, such as Postn-Cre when focusing on activated CFs.

Next, SM22α-Cre is expressed in some immune cells, including phenotypically defined putative HSCs. It was unlike the results of a previous paper^5^, but Zovein et al. observed SM22α-Cre recombination at E11.5 in the aortic-gonado-mesonephros (AGM) region, the origin of HSCs^20^. This is likely to support the results on the recombination in HSCs, but further in vivo repopulating experiments would confirm this conclusion.

Considering the results, we recommend that when examining inflammation models such as myocardial infarction and heart failure with SM22α-Cre, bone marrow transplantation should be performed first.

Finally, in vitro, SM22α-Cre is a good tool to modify genes of murine fibroblasts. However, in BMDMs and other immune cells, we cannot induce SM22α-Cre recombination during adulthood because the RNA level of Tagln is very low, similar to the open RNA-seq data^21^. This may be due to the expression of Transgelin-2, which is one of the homologues of SM22α^22^, in these cells. Transgelin-2, the only transgelin isoform expressed in immune cells, acts as a molecular staple to stabilize the actin cytoskeleton; this function may replace the function of SM22α^23^.

The reason for recombination in immune cells is unclear. In addition to the SM22α-Cre recombination in the AGM region as mentioned above, recent single-cell data about preHSCs may reveal this answer^24^. The data indicated that recombination in immune cells may occur via the endothelial to hematopoietic transition (Supplementary Fig. 3a–c). This effect may be because the downregulation of Erg1, a key transcription factor at the endothelial to hematopoietic transition, upregulates SM22α^25^.

## Materials and methods

### Animals

All animal experiments were performed in accordance with the institutional guidelines of the Institute of Laboratory Animals, Graduate School of Medicine, Kyoto University (Kyoto, Japan), and all experimental protocols were approved by the same institute. The reporting in this manuscript followed the recommendations in the ARRIVE guidelines. For euthanasia, mice were intraperitoneally administrated with a mixture of Medetomidine (0.3 mg/kg body weight), Midazolam (4 mg/kg body weight), and Butorphanol (5 mg/kg body weight). SM22α-Cre mice(Tg (Tagln-cre)1Her/J, Stock# 004746) and dual fluorescent membrane-localized tdTomato/eGFP (mT/mG) mice (B6.129(Cg)-Gt(ROSA)26Sortm4(ACTB-tdTomato, -EGFP)Luo/J, Stock# 007676) were purchased from the Jackson Laboratory. SM22α -Cre mice were bred with flox mT/mG mice to produce mT/mGflox/wt: SM22α-Cre + (SM22mT/mG) mice. For embryos, SM22α -Cre mice were paired overnight with flox mT/mG mice. The morning after mating was considered 0.5 days post-coitus (dpc).

### Preparation of cells

Male and female SM22mT/mG mice, 6–10 weeks of age, were used as the cell source. CFs were isolated as described previously^26^. Media and buffers were prepared according to a previous paper^26^. The descending aortas and inferior venae cavae were cut. The hearts were perfused with EDTA buffer from the right ventricle. The ascending aortas were clamped. The clamped hearts were removed, transferred to a dish containing EDTA buffer, and perfused with EDTA buffer from the left ventricle (LV). The hearts were transferred to a dish of perfusion buffer, and perfused with perfusion buffer from the LV. The hearts were transferred to a dish of collagenase buffer and perfused with collagenase buffer from the LV. The ventricles were transferred to the other dish of collagenase buffer, gently teased apart into pieces, and triturated. Stop solution was added to the cell-tissue cell suspension. The supernatants obtained via gravity settling three times for 10 min in perfusion buffer were collected as nonCMs. The nonCMs were centrifuged at 300×*g* for 5 min, resuspended in DMEM containing 10% fetal bovine serum (FBS) and penicillin–streptomycin (Wako, #168-23191), plated on cell culture dishes and cultured for 6–7 days. Almost all cells were fibroblasts after the culture.

Bone marrow was extracted from the femurs and tibias of euthanized mice and differentiated in bone marrow macrophage differentiation media (RPMI 1640 containing 10% fetal bovine serum (FBS), penicillin–streptomycin, 20 μg/mL recombinant mouse M-CSF (Biolegend, #576404), and 0.1 mM/L 2-mercaptoethanol (Wako, #133-14571)). Seven days after being harvested, BMDMs were stimulated with recombinant mouse TGF-β1 (Biolegend, # 763102) for 1 or 7 days, for RNA or for Flow cytometric analysis, respectively. For RNA analysis, cells were cultured under serum-free media (RPMI +1% Bovine serum albumin) (Merck, #A9418) during stimulation. To investigate whether Cre-recombination occurs during the differentiation process from HSCs to BMDMs, bone marrow cells were harvested and cultured in bone marrow macrophage differentiation media containing with TGF-β.

### Immunostaining

Hearts in 6-week-old mice were perfused with cold phosphate-buffered saline (PBS) and 4% paraformaldehyde, removed, and fixed with 4% paraformaldehyde (PFA) for 3 h. The hearts of Embryos at 17.5 dpc were removed, washed with cold PBS and fixed with 4% PFA for 3 h. The hearts were incubated in 10%, 20% and 30% sucrose diluted in PBS. The samples were then embedded in OCT compound (Sakura Finetek Japan), frozen and stored at − 80°. Cryosections (8 µm thick) were obtained using a Leica cryostat.

For immunofluorescence analysis, sections were first washed with PBS, permeabilized with PBS containing 0.1% Triton-X, and washed with PBS containing 0.1% Tween 20. The sections were then incubated in blocking buffer (PBS containing, 0.1% Tween 20, 1% BSA, and 10% normal donkey serum (Jackson ImmunoResearch, #017-000-121)) for 1 h at room temperature. Primary antibodies diluted in blocking buffer were added and incubated overnight at 4°. The following primary antibodies were used: anti-αSMA (1:100) (Goat, Novus Biologicals, #NB300-978), anti-Vimentin (1:100) (rabbit, Cell Signaling Technology, #5741S), anti-CD68 (1:200) (rat, Bio Rad, #MCA1957GA), and anti-CD31 (1:100) (rabbit, Novus Biologicals, # NB100-2284). Then, the slides were washed three times in PBS containing 0.1% Tween 20 for 5 min each, and incubated with Alexa Fluor 647 (ThermoFisher, #A-31573 or Abcam, #ab150155) or Alexa Fluor Plus 680 (ThermoFisher, #A32860) against the appropriate species (diluted at 1:500) for 1 h at room temperature. The slides were washed three times in cold PBS for 5 min each. Finally, the slides were mounted with VECTASHIELD Antifade Mounting Medium with DAPI (Vector Laboratories, #H-1200-10).

Immunofluorescence images were acquired on an Axio Observer (Carl Zeiss) (5 × objective) or SP8 Falcon (Leica) (63 × objective) and analyzed with Zen software (Carl Zeiss) or LAS X (Leica), respectively.

### Flow cytometric analysis

Single cardiac cell suspensions were generated as described previously^27^. Hearts were perfused with cold PBS and finely minced and digested in Hank’s Balanced Salt Solution (HBSS) with Collagenase 2 (500 U/ml) (Worthington Biochemical, #LS004176) for 30 min at 37 °C. Next, the hearts were digested in HBSS with Collagenase/Dispase (1 mg/mL) (Merck, #11097113001) for 20 min at 37 °C. To deactivate the enzymes, the samples were washed with cold HBSS. The solutions were passed through a 40 μm cell strainer (Corning, #352340). Red blood cell lysis was performed with ACK lysis buffer (0.16 M ammonium chloride, 10 mM Potassium bicarbonate and 0.1 mM EDTA). The samples were washed with FACS buffer (HBSS with 25 mM 4-(2-hydroxyethyl)-1-piperazineethanesulfonic acid (HEPES), 2% FBS and 2 mM ethylenediaminetetraacetic acid (EDTA)) and resuspended in 300 μL of FACS buffer. Heart samples were blocked with TruStain FcX Plus (0.5 μL/100 μL) (Biolegend, #156604) for 5 min at 4°.

Peripheral blood samples were collected from the inferior vena cava of anesthetized mice using a heparin-contained syringe. Red blood cell lysis was performed with ACK lysis buffer. The samples were washed with FACS buffer and resuspended. Peripheral blood samples were blocked with TruStain FcX Plus for 5 min at 4°.

Bone marrow cells were obtained by flushing femurs and tibias with RPMI supplemented with 25 mM HEPES and 10% FBS. The suspensions were passed through a 40 μm cell strainer. After the red blood cells were lysed, the samples were washed with FACS buffer and resuspended.

BMDMs were collected after harvesting as described before and were blocked with TruStain FcX Plus for 5 min at 4°.

Cells were stained with monoclonal antibodies at 4 °C for 20 min in the dark. The samples were washed twice, and the final resuspension was made in 500 μL of FACS buffer. 7-AAD was used to exclude dead cells. Flow cytometric analysis was performed on BD FACS ARIAII platforms. Complete lists of antibodies and flow cytometry gating strategies are provided in Supplementary Tables 1 and 2, respectively.

### RNA extraction and qRT–PCR

Total RNA was isolated and purified using TRIzol reagent (ThermoFisher), and cDNA was synthesized using ReverTra Ace qPCR RT Master Mix with gDNA Remover (TOYOBO, #FSQ-301) in accordance with the manufacturer's instructions. For quantitative real-time PCR (qRT–PCR), specific genes were amplified using 40 cycles with Thunderbird SYBR qPCR mix (Toyobo, #QPS-201) and StepOnePlus (ThermoFisher). Expression was normalized to the housekeeping gene *18rS*. Gene-specific primers are described as follows:

18rS forward, CTCAACACGGGAAACCTCAC; 18rS reverse, AGACAAATCGCTCCACCAAC; Tagln forward, CAACAAGGGTCCATCCTACGG; Tagln reverse, ATCTGGGCGGCCTACATCA; Acta2 forward, TGACGCTGAAGTATCCGATAGA; Acta2 reverse, CGAAGCTCGTTATAGAAAGAGTGG; Col1a1 forward, AATGGCACGGCTGTGTGCGA; and Col1a1 reverse, AACGGGTCCCCTTGGGCCTT.

### Statistical analysis

For the Flow cytometric analysis, the lines represent the means and standard error of the samples. Differences between two groups were compared by Student’s t test as a parametric comparison test. For the RT–PCR experiments, one-way ANOVA followed by Tukey’s test was performed for multiple comparisons. The bars represent mean of the samples. The analysis and plots were generated using the ggplot2 package and R software. A P value < 0.05 was considered statistically significant.

## Supplementary Information

Supplementary Figures.

Supplementary Information 1.

## Data Availability

All data generated or analyzed during this study are included in this published article and its supplementary information file.
