# Author Correction: Smooth muscle protein 22α-Cre recombination in resting cardiac fibroblasts and hematopoietic precursors

**DOI:** 10.1038/s41598-022-26447-w

**Published:** 2022-12-20

**Authors:** Shinya Ikeda, Sachiko Sugioka, Takeshi Kimura, Noboru Ashida

**Affiliations:** 1 Department of Cardiovascular Medicine, Graduate School of Medicine, Kyoto University, 54 Kawahara-cho, Shogoin, Sakyo-ku, Kyoto, 606-8507 Japan; 2 Hirakata Kohsai Hospital, Osaka, Japan

Correction to: *Scientific Reports* 10.1038/s41598-022-15957-2, published online 07 July 2022

The original version of this Article contained an error in TGF-β units in Figure 4 (d)-(g) labels, where

“μg/ml”

now reads:

“ng/ml”

The original Figure 4 and accompanying legend appear below.

**Figure 4:**
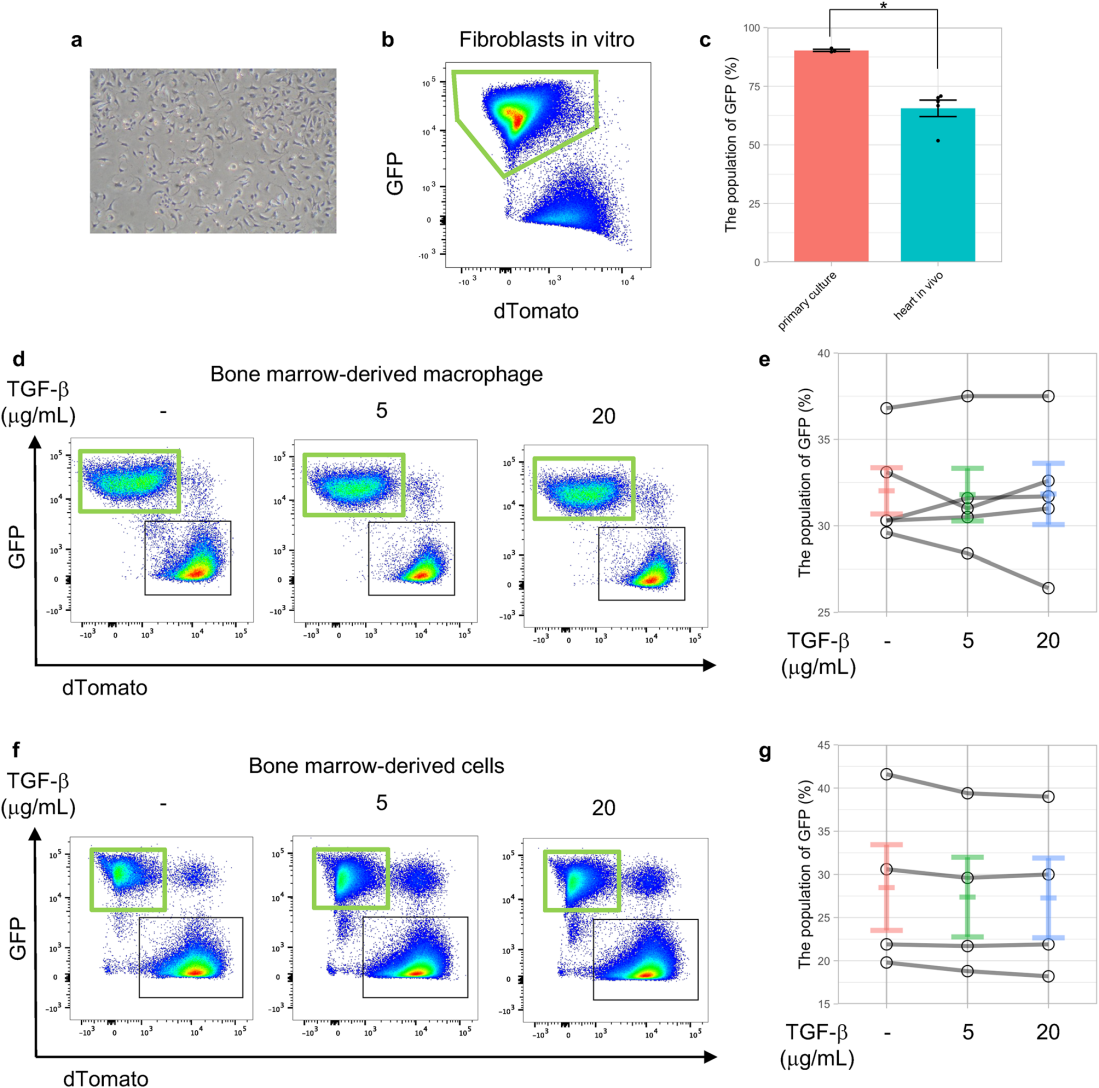
SM22α-Cre recombination in vitro. (**a**) A representative bright field image of cultured fibroblasts. (**b**) GFP expression in fibroblasts in vitro. (**c**) GFP expression in fibroblasts in vitro (n = 3) and in vivo (n = 5). *p value < 0.01, evaluated by Student’s t test. (**d**) GFP expression in BMDMs with TGF-β stimulation. (**e**) No additional SM22α-Cre recombination in BMDMs with TGF-β stimulation, which increases SM22α expression. (**f**) GFP expression in bone marrow-derived cells with TGF-β stimulation. (**g**) No additional SM22α-Cre recombination in bone marrow-derived cells during differentiation from phenotypically defined putative HSCs to macrophages with TGF-β stimulation.

Additionally, the same error occurred in Supplementary Information Figure 2.

Lastly, the article omitted to mention that BMDMs were cultured under serum-free media. Consequently, in Materials and methods, under the subheading ‘Preparation of cells’,

now reads:

"For RNA analysis, cells were cultured under serum-free media (RPMI +1% Bovine serum albumin) (Merck, #A9418) during stimulation.”

The original Article and Supplementary Information file have been corrected.

