# Supplementary figures and images for: Smooth muscle protein 22α-Cre recombination in resting cardiac fibroblasts and hematopoietic precursors
Source: Sci Rep. 2022 Jul 7;12:11564. doi: 10.1038/s41598-022-15957-2 (PMC9263136; doi:10.1038/s41598-022-15957-2)

Supplementary Figure1

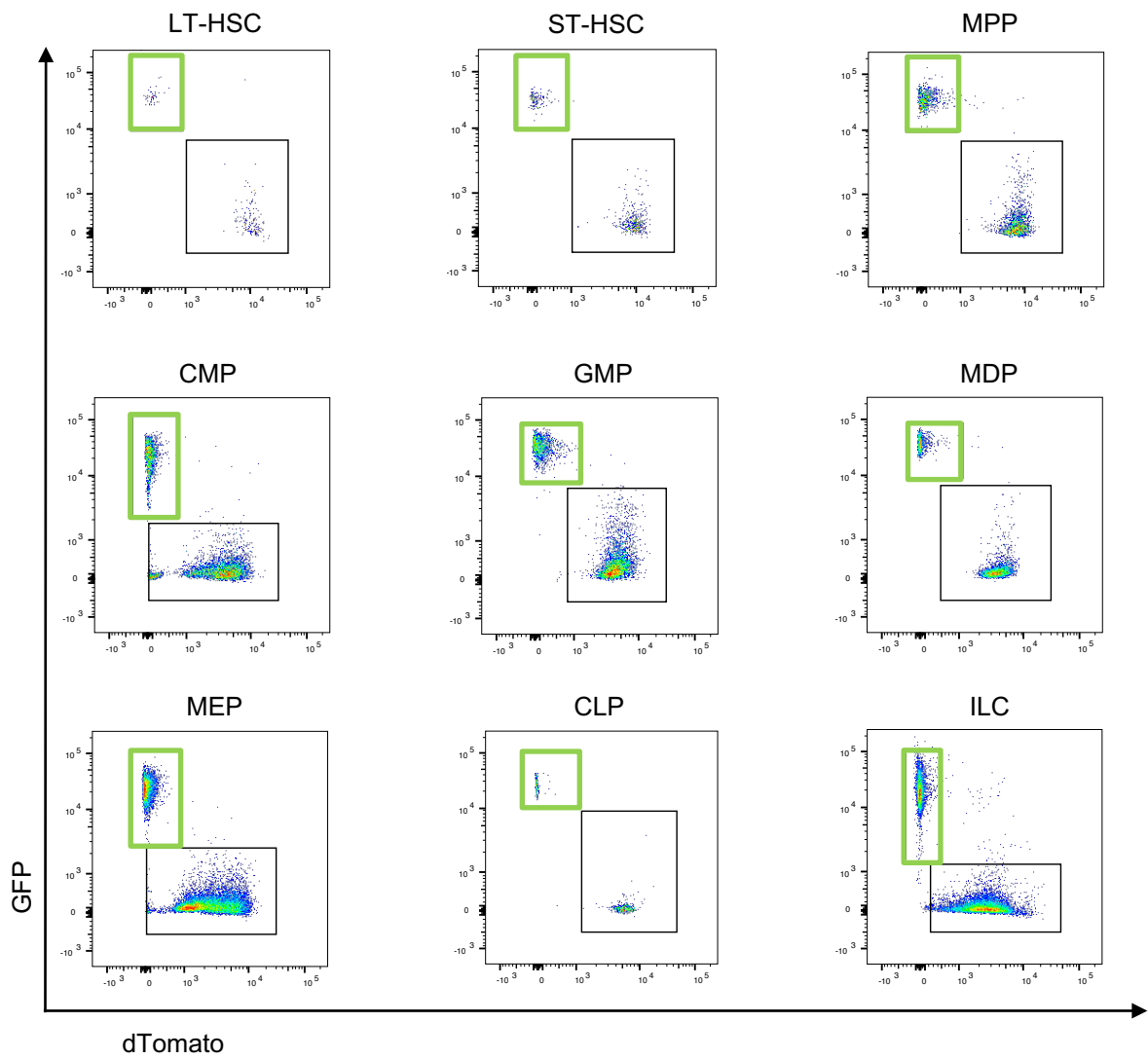

Supplementary Figure 2

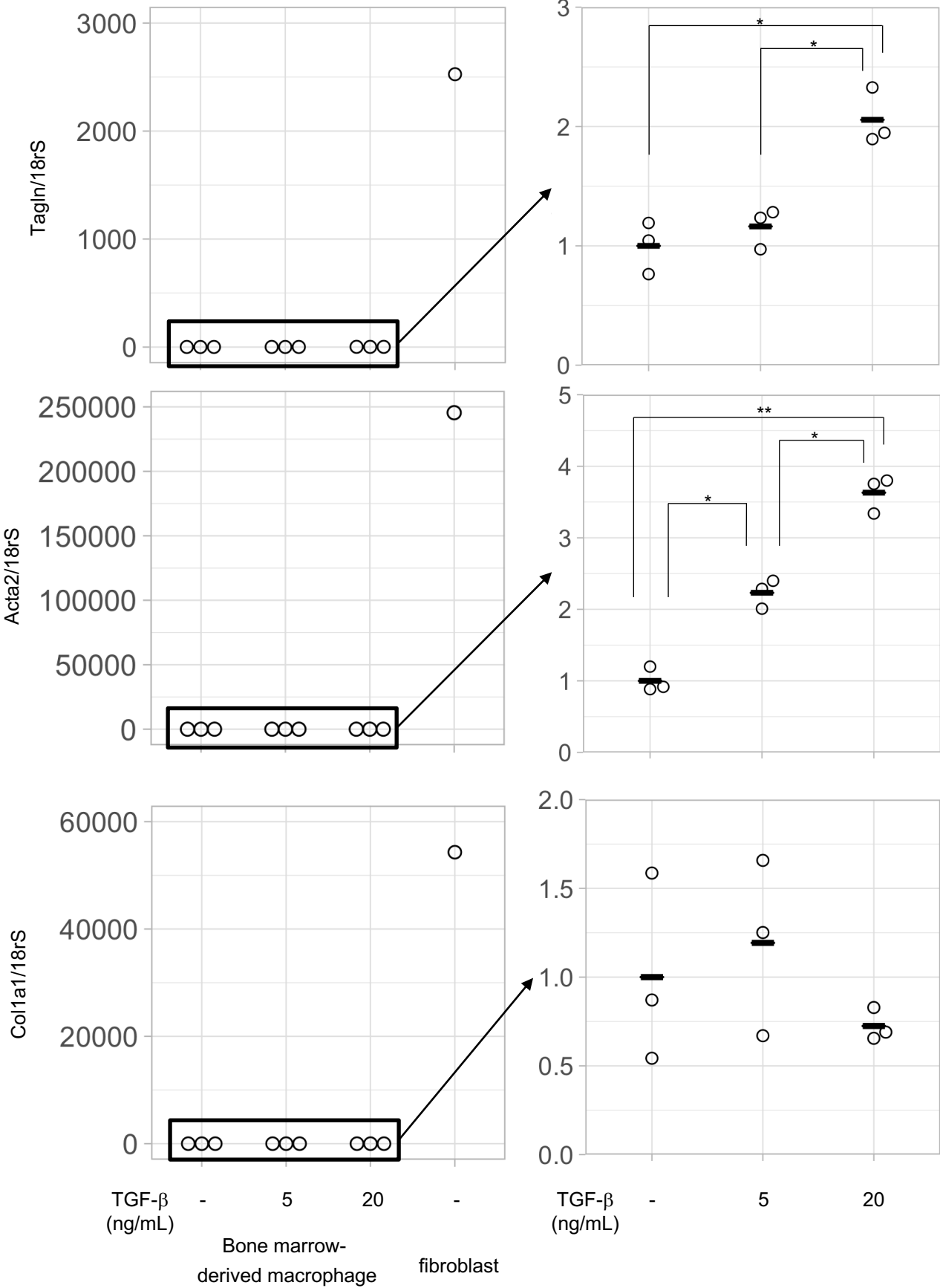

Supplementary Figure3

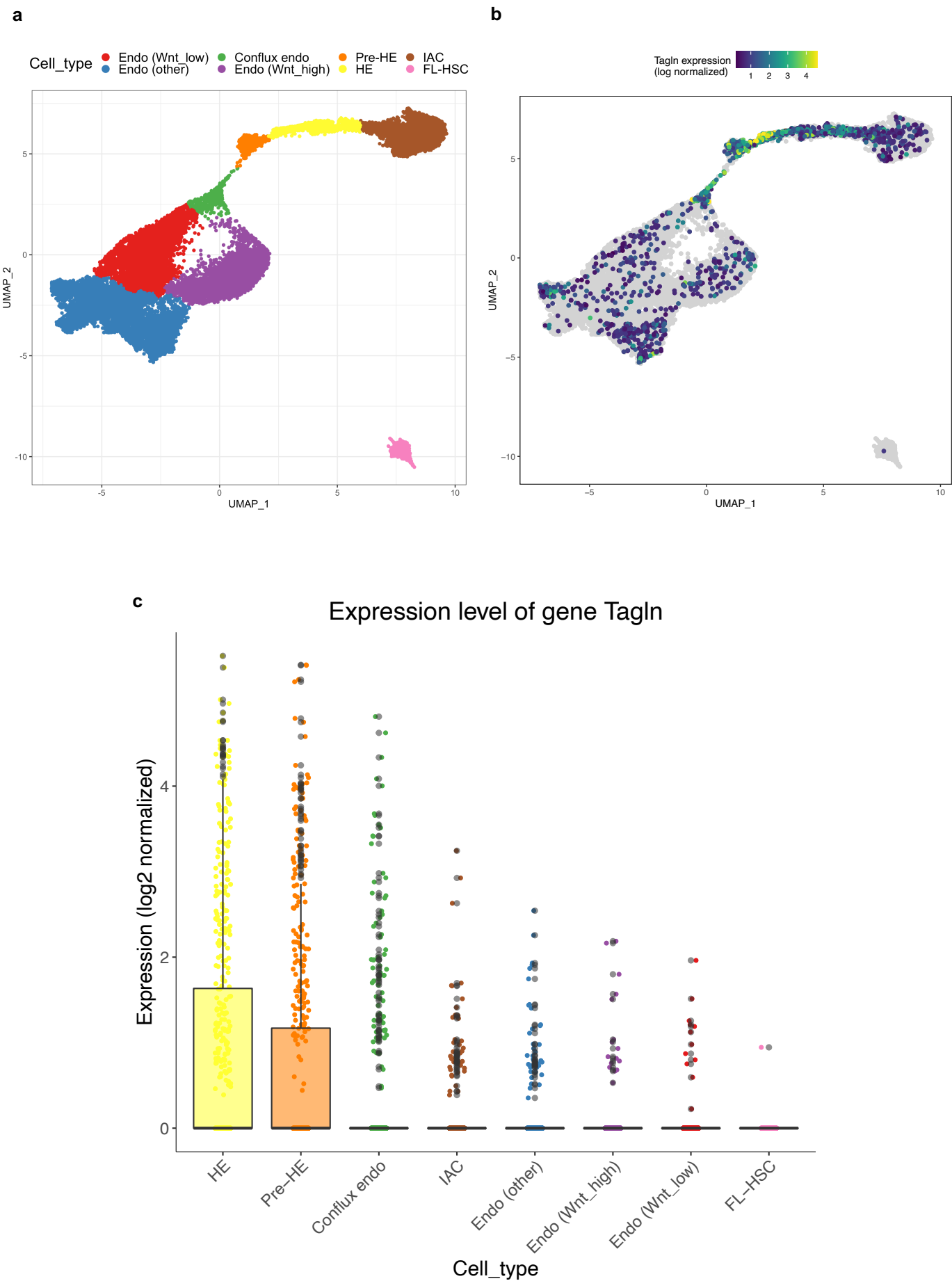

Supplement: Supplementary file 1 — Supplementary Figures. [file 41598_2022_15957_MOESM1_ESM.pdf]
