# Supplementary material for: Smooth muscle protein 22α-Cre recombination in resting cardiac fibroblasts and hematopoietic precursors
Source: Sci Rep. 2022 Jul 7;12:11564. doi: 10.1038/s41598-022-15957-2 (PMC9263136; doi:10.1038/s41598-022-15957-2)
Supplement: Supplementary file 2 — Supplementary Information 1. [file 41598_2022_15957_MOESM2_ESM.docx]

**Supplementary Figure 1. Representative images of the GFP-positive populations among each cell type in SM22mTmG’s bone marrow, as analyzed by flow cytometry.**

LT-HSC: phenotypically defined putative long-term hematopoietic stem cell, ST-HSC: phenotypically defined putative short-term hematopoietic stem cell, MPP: multipotent progenitor, CMP: common myeloid progenitor, GMP: granulocyte macrophage progenitor, monocyte-macrophage dendritic cell progenitor, MEP: megakaryocyte-erythrocyte progenitors, CLP: common lymphoid progenitor, ILC: innate lymphoid cell.

**Supplementary Figure 2. The downstream genes of TGF-**β **signaling in BMDMs.**

Tagln, Acta2 and Col1a1 levels in BMDMs were much lower than those in cultured fibroblasts, although TGF-β increased Tagln and Acta2 levels in BMDMs. For statistical analysis, one-way ANOVA followed by Tukey’s test was performed for multiple comparisons. *p value < 0.01, **p value < 0.001.

**Supplementary Figure 3. Single cell RNA-sequencing data on Tagln (SM22α) expression in the endothelial to hematopoietic transition (EHT).**

**(a)** UMAP of the continuous EHT trajectory in selected cell populations labeled in the article. **(b)** Tagln expression in EHT. **(c)** Tagln expression in each cell cluster. Tagln was detected during EHT. Endo: endothelial, HE: hemogenic endothelial, IAC: intra-arterial cluster, FL-HSC: Fetal liver hematopoietic stem cell

**Supplementary Table 1. The list of antibodies and material used in flow cytometry.**

| Antigen | Clone | Fluorescent dye | Company | Catalog number |
| --- | --- | --- | --- | --- |
| CD31 | 390 | PE-Cyanine7 | BioLegend | 102418 |
| CD31 | MEC13.3 | BV421 | BD biosciences | 562939 |
| CD45 | 30-F11 | BUV395 | BD biosciences | 564279 |
| CD45 | 30-F11 | APC-Cyanine7 | BioLegend | 103116 |
| PDGFRa | APA5 | APC | BioLegend | 135908 |
| CD11b | M1/70 | BUV737 | BD biosciences | 741722 |
| CD11b | M1/70 | BV605 | BD biosciences | 563015 |
| Ly6G | 1A8 | BV605 | BioLegend | 127639 |
| CD64 | X54-5/7.1 | PE-Cyanine7 | BioLegend | 139314 |
| Ly6C | HK1.4 | APC-Cyanine7 | BioLegend | 128025 |
| MHC2 | M5/114.15.2 | BV605 | BioLegend | 107639 |
| CCR2 | 475301 | APC | R&D Systems | FAB5538A |
| CD3e | 145-2C11 | APC | BioLegend | 100311 |
| B220 | RA3-6B2 | BV421 | BioLegend | 103239 |
| CD115 | AFS98 | PE-Cyanine7 | BioLegend | 135523 |
| CD115 | AFS98 | BUV395 | BD biosciences | 750886 |
| Lineage Cockatil |  | BV421 | BioLegend | 133311 |
| c-Kit | 2B8 | APC-Cyanine7 | BioLegend | 105825 |
| Sca1 | D7 | BV605 | BioLegend | 108133 |
| CD48 | HM48-1 | APC | BioLegend | 103411 |
| CD150 | TC15-12F12.2 | PE-Cyanine7 | BioLegend | 115913 |
| CD34 | RAM34 | eFluor 660 | ThermoFisher | 50-0341-82 |
| CD16/32 | 93 | PE-Cyanine7 | BioLegend | 101317 |
| CD135 | A2F10 | APC | BioLegend | 135309 |
| CD127 | A7R34 | PE-Cyanine7 | BioLegend | 135013 |
| 7-AAD |  |  | BioLegend | 420404 |

**Supplementary Table 2. Flow cytometry gating.**

| **Cell type** | **Gating** |
| --- | --- |
| **Endothelial cell** | CD45-, CD31+ |
| **Fibroblast** | CD45-, CD31-, PDGFRa+ |
| **Macrophage** | CD45+, CD11b+, CD64+, Ly6c low |
| **Tissue resident macrophage** | CD45+, CD11b+, CD64+, Ly6c low, CCR2-, MHC2 low |
| **Macrophage MHC2 high** | CD45+, CD11b+, CD64+, Ly6c low, CCR2-, MHC2 high |
| **Macrophage CCR2+** | CD45+, CD11b+, CD64+, Ly6c low, CCR2+ |
| **Neutrophil** | CD45+, CD11b+, Ly6G+ |
| **T-cell** | CD45+, CD11b-, CD3e+ |
| **B-cell** | CD45+, CD11b-, B220+ |
| **Monocyte** | CD45+, CD11b+, Ly6G-, CD115+ |
| **LT-HSC** | Lineage-, Sca-1+, c-Kit+, CD150+, CD48- |
| **ST-HSC** | Lineage-, Sca-1+, c-Kit+, CD150-, CD48- |
| **MPP** | Lineage-, Sca-1+, c-Kit+, CD150-, CD48+ |
| **CMP** | Lineage-, Sca-1-, c-Kit+, CD34+, CD16/32- |
| **GMP** | Lineage-, Sca-1-, c-Kit+, CD34+, CD16/32+, CD115- |
| **MDP** | Lineage-, Sca-1-, c-Kit+, CD34+, CD16/32+, CD115+ |
| **MEP** | Lineage-, Sca-1-, c-Kit+, CD34-, CD16/32- |
| **CLP** | Lineage-, Sca-1 low, c-Kit low, CD135+, CD127+ |
| **ILC** | Lineage-, Sca-1+, c-Kit- |

LT-HSC: phenotypically defined putative long-term hematopoietic stem cell, ST-HSC: phenotypically defined putative short-term hematopoietic stem cell, MPP: multipotent progenitor, CMP: common myeloid progenitor, GMP: granulocyte macrophage progenitor, monocyte-macrophage dendritic cell progenitor, MEP: megakaryocyte-erythrocyte progenitors, CLP: common lymphoid progenitor, ILC: innate lymphoid cell.

Supplementary Methods

**Dataset**

For Supplemental Figure 3, we used the single-cell RNA sequencing and single- cell assay for transposase-accessible chromatin sequencing dataset provided by Zhu et al. We analyzed the dataset in the same way as Zhu did.
